# Transformation of metabolism with age and lifestyle in Antarctic seals: a case study of systems biology approach to cross-species microarray experiment

**DOI:** 10.1186/1752-0509-4-133

**Published:** 2010-09-29

**Authors:** Andrey Ptitsyn, Amber Schlater, Shane Kanatous

**Affiliations:** 1 Colorado State University Center for Bioinformatics, Fort Collins, CO 80523, USA; 2 Colorado State University Department of Biology, Fort Collins, CO 80523, USA

## Abstract

**Background:**

The metabolic transformation that changes Weddell seal pups born on land into aquatic animals is not only interesting for the study of general biology, but it also provides a model for the acquired and congenital muscle disorders which are associated with oxygen metabolism in skeletal muscle. However, the analysis of gene expression in seals is hampered by the lack of specific microarrays and the very limited annotation of known Weddell seal (*Leptonychotes weddellii*) genes.

**Results:**

Muscle samples from newborn, juvenile, and adult Weddell seals were collected during an Antarctic expedition. Extracted RNA was hybridized on Affymetrix Human Expression chips. Preliminary studies showed a detectable signal from at least 7000 probe sets present in all samples and replicates. Relative expression levels for these genes was used for further analysis of the biological pathways implicated in the metabolism transformation which occurs in the transition from newborn, to juvenile, to adult seals. Cytoskeletal remodeling, WNT signaling, FAK signaling, hypoxia-induced HIF1 activation, and insulin regulation were identified as being among the most important biological pathways involved in transformation.

**Conclusion:**

In spite of certain losses in specificity and sensitivity, the cross-species application of gene expression microarrays is capable of solving challenging puzzles in biology. A Systems Biology approach based on gene interaction patterns can compensate adequately for the lack of species-specific genomics information.

## Background

The number of species that come under the spotlight of biological research by far exceeds the number of model organisms for which specific gene expression tools are available. Analysis of gene expression patterns in non-model species does not merely serve our inexhaustible curiosity about our planetary companions. Understanding adaptation in other species provides valuable clues for understanding our own physiology, molecular mechanisms of disease, and potential cures. One remarkable example of this comes from a seemingly unlikely source: the Weddell seal.

Weddell seals (*Leptonychotes weddellii*) inhabit the cold waters around the Antarctic continent. During their lifetime, seals experience a cardinal transformation in lifestyle and physiology. Pups are born on fast ice and spend the first months of their life as land animals. Then, while juvenile seals are learning to dive, they have a lifestyle similar to the harbor seals (*Phoca vitulina*) which inhabit the shallow coastal waters of the Northern hemisphere. Later in life, however, Weddell seals develop into elite, deep long duration divers. During their transition from a land to a marine lifestyle, seal physiology undergoes a series of changes: an increase in the aerobic efficiency of the skeletal muscles, a shift towards fatty acid catabolism for aerobic ATP production, enhanced oxygen storage, improved diffusion capacity, and reduced dependency on blood-borne oxygen and metabolites (e.g. decreased capillary density) compared with terrestrial mammals [1]. It has been reported that the seals also undergo transformation in muscle fiber composition and mitochondrial density from a state similar to athletic terrestrial animals to that of sedentary terrestrial animals of compatible size [1].

Further understanding of this transition requires both tracing the change in activity of individual genes and a broad overview of scores of different genes. A microarray survey of activity for a few thousand genes in each sample provided the needed boost for the study. However, the application of microarray technology, almost routine in other areas, presents a series of challenges for the study of Weddell seals. This is because there is no microarray specifically developed for this or any other related species of seal. There is also no reference genome sequence available at this point. The development of custom arrays from the Expressed Sequence Tag (EST) libraries is possible, but this process is expensive and time consuming. There are remarkable examples of gene expression using custom microarrays for dolphins [2-4]. However, the greatest obstacle for using custom arrays is the lack of functional annotation for ESTs. There is also no specific information on gene interaction for this species. It is reasonable to believe that canonic biological pathways on Weddell seal muscle are sufficiently similar to those of more closely studied model organisms; such as mice, rats or humans. Thus, information on major metabolic pathways in general can be used as a guide to understanding specific changes in seal physiology; if only it were possible for expression patterns in seal muscles to be mapped to the database of metabolic pathways of humans.

The idea of cross-species hybridization is almost as old as microarray technology (see [5] for review). It has been used in many different studies on organisms for which a specific microarray platform were not available: examples range from cross-hybridization between different species of salmon and trout using salmon-derived microarray [6] to the application of a tomato microarray to study eggplants [7]. Cross-species applications of microarrays can be successfully applied for the analysis of evolutionary relations and are a boon for naturalists who are interested in functional genomics. Reproducibility of microarray hybridization seems to correlate with the phylogenetic distance in branching species[8]. However, the successful identification of a few differentially expressed genes is insufficient for understanding the nature and the mechanism of metabolic transformation associated with age and lifestyle transition in Weddell seals. We need to acquire a broad scope of gene expression patterns and to identify the molecular structures and functions implicated in deep diving adaptation.

## Methods

### Animals

Twenty-four newborn Weddell seal pups (age 3-5 weeks, mean mass 75 ± 3 kg) were captured over three field seasons (October-December of 2002, 2005, and 2006) by using mild physical restraints or a purse string net near the pupping colonies in McMurdo Sound, Antarctica. Likewise, eighteen juvenile (age 1-2 years, mean mass 120 ± 5 kg) and twenty-six adult Weddell seals (age 7+ years, mean mass 385 ± 13 kg) were captured with a purse string net along natural tidal cracks in McMurdo Sound. The ages of all the seals were determined from flipper tags with the data provided by personal communication with R. Garrott, J. Rotella, and D. Siniff (NSF grant OPP-0225110). The seals were mildly sedated with ketamine (1.5 mg/kg, [1]), weighed with a hanging digital scale (accuracy ± 0.5 kg), and muscle biopsies taken under local anesthesia. In order to standardize our sampling, all biopsies were taken from the same location in all age classes: the mid-belly of the muscle (1/3 of the body length from the tail).

### Muscle biopsies

Muscle samples of approximately 50 mg each were collected with a 6-mm biopsy cannula (Depuy, Warsaw, Indiana) from the swimming (M. longissimus dorsi) muscle of the seals. Muscle samples were placed in Qiagen "RNA later" and refrigerated overnight. The samples were then frozen in liquid nitrogen and stored at -80°C until analysis.

### RNA isolation

Samples intended for micro-array analysis were sorted into Tripure (Gibco) to maximize RNA recovery. Total RNA was prepared following standard protocols then treated with Dnase I using the RNeasy column purification system (Qiagen). A portion of the total RNA was hybridized to micro-array chips (Affymetrix Human 2.0 gene chip) and analyzed using Affymetrix software.

### Overview of the analysis pipeline

**Figure 1:**
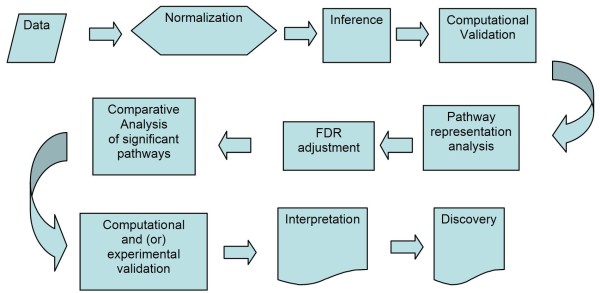
**Overview of the analysis pipeline**. The pipeline includes most of the standard analysis steps, but has a few important differences. We extend the pipeline to maximize the advantage of pathway analysis. The genes important for understanding the biological processes involved in metastatic transformation are selected not solely by the difference in signal emitted by microarray probes. Instead, we concentrate on the "group behavior" of genes.

The general overview of the analysis pipeline is given in Figure 1. Our pipeline includes most of the standard analysis steps, with a few important differences. First of all, we extended the pipeline to maximize the advantage of pathway analysis. In addition, the genes important for understanding the biological processes involved in metastatic transformation were not selected based solely on the difference in the signal emitted by the microarray probes. Instead, we concentrated on the "group behavior" of the genes, including their ability to interact and the pre-existing annotation which places the genes into the same biological pathway, thereby linking them to the same cellular function. Thus, the inference (or selection of potentially meaningful differentially expressed genes) was done using very liberal selection criteria and was not adjusted for multiple testing. We selected a large list of potentially differential genes with the potential to contain a large number of false-positives. Then we selected biological pathways, molecular functions, and GO terms which were statistically over-represented in the initial intensity-based list. This procedure is part of the standard toolbox in the GeneGo MetaCore software package. The benefits of using pathways and ontological analyses of the microarray data have been presented previously [9,10]. The significance of biological pathways was estimated by using a variation of Fisher's exact test as implemented in GeneGo's MetaCore software suite, and then adjusted for multiple testing using Benjamini-Hochberg FDR analysis (which is also a built-in function of the GeneGo Metacore software). Single genes that do not map into any statistically significant pathway (i.e. missing all regulators, downstream targets, ligands, and other components necessary for a functional molecular mechanism) may be still considered to be significant if they are reproducible and can be independently validated in additional experiments. Our approach relies on the collective effects of groups of genes which are interlinked by functional relationships. This approach may be inapplicable to a number of genes which lack information on function, regulation, and interaction with other genes.

### Pre-processing and Normalization

The summation of intensity values and the determination of presence/absence calls were performed using the DChip2006 software http://www.dchip.org {Cheng Li, Wing Hung Wong 2001). In addition, the data were normalized using a quantile algorithm similar to the one described by Bolstad et al. [11]: $x_{norm}=F_{2}^{-1}(G(x))$ where *F *is the distribution function of the actual sample, and *G *is the reference distribution function. Reference distribution is an average of sample distribution for the entire data set. Bolstad et al. suggest, but do not elaborate on, an additional smoothing procedure for the sample distribution. In the case of remote cross-species hybridization we observed fewer spots/probesets with a detectable signal and large numbers of probesets emitting a signal no higher than the background noise level. The actual reference distribution is built on a relatively small number of meaningful probesets and by using a very limited number of replicates. Under such circumstances smoothing becomes important, so to ensure smoothing we used a seven-point Savitzky-Golay algorithm [12]. We applied our own C++ software for normalization, which is available from A. Ptitsyn upon request. Box-plots for pre-normalized and normalized expression value distributions are shown in the Supplemental Figure 1 (inside Additional File 1).

### Preliminary selection of differentially expressed genes

A set of differentially expressed genes was selected using the University of Pittsburgh Gene Expression Data Analysis suite (GEDA, http://bioinformatics.upmc.edu/GE2/GEDA.html). For selection, we applied the standard J5 metric with threshold 4 and optional 4 iterations of the Jackknife procedure to reduce the number of false-positive differential genes [13]. This step corresponds to the "Computational Validation" step of the pipeline in Figure 1. Both the J5 metric and the threshold parameter are standard pre-set values recommended by the developers. We did not attempt to estimate the confidence level of individual genes. In addition, we didn't use J5 as a statistical test, but as a selection procedure for providing a shortlist of genes which deviated from the expected average value and were enriched with differential genes. The MA plot showing the selected differential genes is presented in Supplemental Figure 2 (inside Additional File 1). The complete annotated lists for the analyzed data sets are given in the Supplemental Table 1 (inside Additional File 1).

**Table 1:** Pathways significantly over-represented in muscle samples of Weddell seals hybridized on Human U133 expression array.

Map	Map Folders	Cell process	p-Value	Objects	
				present	total
Cytoskeleton remodeling	Regulatory processes/Cytoskeleton remodeling		1.74E-10	41	83

Cytoskeleton remodeling, TGF, WNT and cytoskeletal remodeling	Regulatory processes/Cytoskeleton remodeling		2.60E-07	40	98

Translation, Regulation of translation initiation	Regulatory processes/Translation	translation	2.89E-07	14	19

* Cytoskeleton remodeling, Role PKA in cytoskeleton reorganisation	Protein function/Kinases Regulatory processes/Cytoskeleton remodeling	protein kinase cascade	6.50E-07	18	30

* Signal transduction, PKA signaling	Protein function/G-proteins/GPCR Protein function/Kinases Protein function/Second messenger	second-messenger-mediated signaling, protein kinase cascade, G-protein coupled receptor protein signaling pathway	1.27E-06	15	23

Cell adhesion, Histamine H1 receptor signaling in the interruption of cell barrier integrity	Protein function/G-proteins/GPCR Regulatory processes/Cell adhesion	G-protein coupled receptor protein signaling pathway, cell adhesion	1.54E-06	20	37

Cytoskeleton remodeling, Regulation of actin cytoskeleton by Rho GTPases	Protein function/G-proteins/RAS-group Regulatory processes/Cytoskeleton remodeling	small GTPase mediated signal transduction	1.98E-06	14	21

Transcription, Formation of Sin3A and NuRD complexes and their role in transcription regulation	Regulatory processes/Transcription	transcription	2.81E-06	15	24

Development, Role of HDAC and calcium/calmodulin-dependent kinase (CaMK) in control of skeletal myogenesis	Protein function Second messenger Protein function Transcription factors Regulatory processes Development Myogenesis	second-messenger-mediated signaling, transcription, response to extracellular stimulus	4.22E-06	22	45

Only first 10 pathways shown, the complete table is given is Additional File 1. The analysis is performed using GeneGo MetaCore software.

### Functional annotation and pathway analysis

Analysis of biological pathways was performed using the MetaCore software (GeneGo Inc.) licensed through the Colorado State University Center for Bioinformatics.

### Validation of Microarray expression data

**Figure 2:**
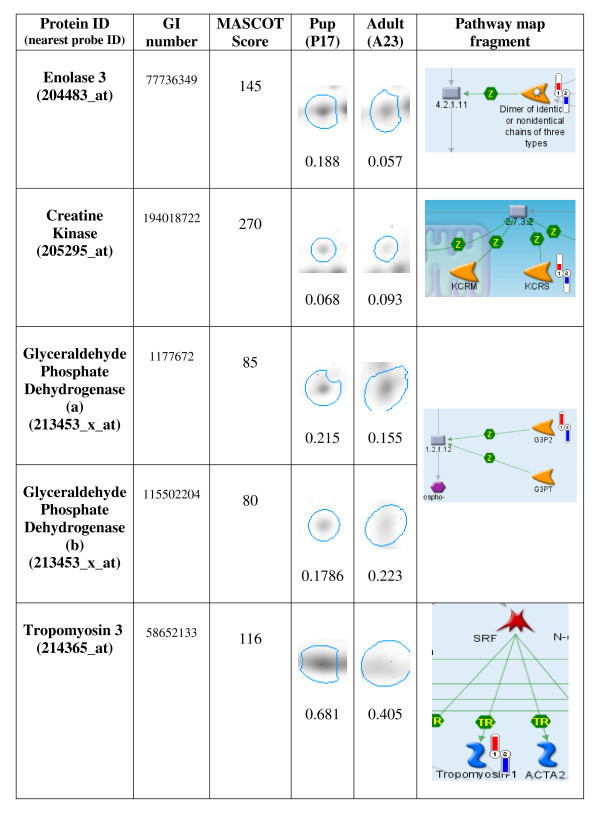
**Protein Spot Identifications in Support of Microarray Data**. Larger, darker spots indicate relatively higher expression. Columns four and five show the spots and quantified median spot density of corresponding proteins. Fragments of Genego pathway maps in the last column indicate direction and scale of expression derived from the microarray data (1-newborns, 2-adults, red indicator in upper direction signifies higher levels of expression, blue indicator directed down signifies lower expression).

Five protein spots identified from the primary swimming muscle (*Longissimus dorsi*) in pup and adult 2 D gels match trends shown in microarray data. The results are presented in Figure 2. The samples are taken from two animals not used in microarray study. Each identification includes the accession number for the MASCOT database and the MASCOT statistical score. Spot densities were quantified using Delta2 D (version 3.6) software.

## Results and discussion

Since the development and application of species-specific custom arrays is, in our case, impractical, and since deep transcriptome analysis with next-generation sequencing technology is financially prohibitive; the only option for large-scale transcription profiling is cross-species hybridization using one of the existing microarray platforms. Among species for which gene expression microarrays are available on the market, the nearest evolutionary neighbor to the Weddell seal is the dog (*Canis familiaris*). However, there are some obvious disadvantages in using, for example, Affymetrix canine expression microarrays in our study. First, there is no reason to expect a high degree of conservation among seal mRNAs and Affymetrix canine probe sequences. Large numbers of Affymetrix microarray probes are derived from target sequences, which in turn are derived from EST clusters. It is widely accepted that expression microarrays, including the Affymetrix canine GeneChip, are biased towards the representation of the 3' untranslated region (UTR). Unlike exons, UTRs, and 3' UTR in particular, are less conserved and tend to diverge, even in closely related species, with a greater divergence in distantly related dogs and seals. Second, *Canis familiaris *is not a popular model organism for most molecular biology studies and little specific information is available about canine gene function, regulation, and interaction. The available functional annotations for gene products represented on the Affymetrix canine microarray are modest compared to mouse or human microarrays. The study conducted with canine microarrays would still rely on the gene interaction and biological pathways data acquired in studies on mice, rats, and humans; as well as the pathway databases for human and most common model organisms. Such a study would also have to rely on a hybridization signal from the subset of probes targeting the more conservative coding section of the genes represented on the microarray. The annotation of human expression arrays is far superior, so why not use the microarrays for these model organisms directly? There is a certain risk that, between distantly related species, there might be an insufficient number and length of conservative sequences to provide an informative signal. However, the list of differentially expressed genes for which cross-species hybridization is successful would be directly admissible to the latest pathway analysis software.

The Affymetrix U133 human expression microarray has yielded in excess of 10,000 probesets (transcripts) called "present" by the DChip program on each chip. As many as 7,664 probesets were present on all the chips used for the experiments. For further analysis we used only genes (probesets) present on all chips. Since we have a very limited number of replicates and hybridized evolutionally divergent mRNA on a human chip we expect a significant occurrence of imperfect hybridization and cross-hybridization. In cases where the signal is low it is hard to determine whether the gene is inactive, expressed at a low level, or simply does not match the probes which are designed for human genes. In this case a standard Affymetrix "absent" call may indicate either absence of a particular gene product or insufficient affinity of the probe due to inter-species differences. Thus, limiting the scope to only probes which perform well and which have a quantifiable signal is a reasonable sacrifice in order to produce more meaningful and reproducible results.

The immediate observation from the preliminary survey of functional annotation and ontology for the detected expressed genes is that, at the very least, the pattern of gene expression is typical for a skeletal muscle tissue. Our previous research has found a significant increase in Type IIA fibers, changes in myoglobin concentration and aerobic efficiency in the skeletal muscles of adult seals as compared to pups and juveniles. In addition, we have shown that these adaptations are associated with changes in calcium signaling [1]. For the pilot "proof of concept" hybridization on the human U133 microarray we have used 3 biological replicates of adult muscle tissue. Further experiments which compare patterns of gene expression between different ages are also dominated by the genes and pathways typical for skeletal muscle; which supports the thesis that seal mRNAs not only hybridize on human microarrays, but reflect the underlying biology of the tissue samples (see Table 1).

**Figure 3:**
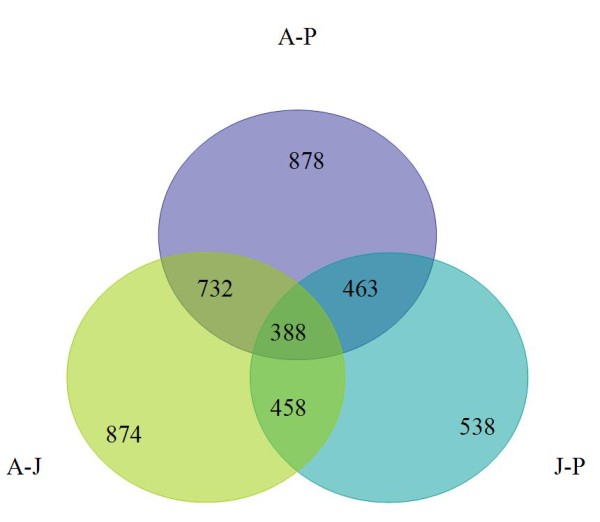
**Numbers of genes differentially expressed between age groups of Wedell seals**. A-J corresponds to adult vs. juvenile; A-P corresponds to adult vs. newborn pups and J-P corresponds to juvenile vs. newborn comparisons. The largest number of differentially expressed genes was found between the adults and pups, followed by the adults and juveniles and finally the smallest number of differentially expressed genes between the pups and juveniles.

**Figure 4:**
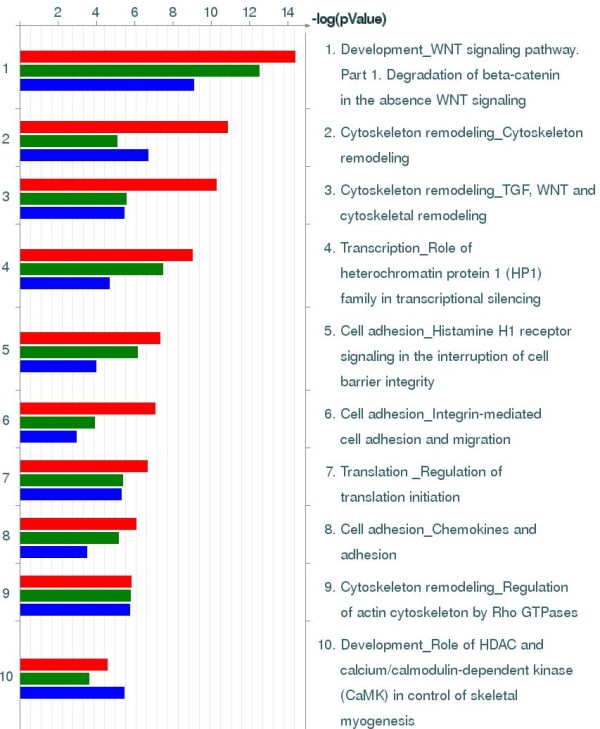
**Top most prominent biological pathways associated with age and lifestyle transformation in skeletal muscle of Weddell seals**. Each bar corresponds to one of the three lists of genes differentially expressed between different age groups. Red bars correspond to adult-juvenile (AJ) comparison; green bars corresponds to adult-newborn comparison (AP) and green bars correspond to newborn-juvenile comparison (PJ).

Analysis of canonic biological pathways, statistically overrepresented among differentially expressed genes, reveals a number of prominent biological pathways. Table 1 shows ten of the most significantly overrepresented pathways (see complete list in Supplemental Table 2, inside Additional File 1). As was expected from the previous studies on the muscle physiology of Weddell seals [1], the group of pathways most affected by age and lifestyle transformation is associated with cytoskeleton remodeling - the production of different types of muscle fibers and related signaling pathways. We have compared pairs of age groups (adults vs. newborn pups, adults vs. juveniles, and juveniles vs. newborn) and generated three lists of differentially expressed genes. These genes were found to be overlapping, with approximately half of the differential genes found on all three lists. This means that, in most cases, the difference in the patterns of gene expression between ages can be explained by the quantitative difference in activity, rather than the turning on or off behavior of certain genes or pathways. This observation is in agreement with previous studies and speaks in favor of gradual adaptation rather than the complete restructuring of metabolism with age. A Venn diagram of differential genes is presented in Figure 3. The alternative analysis of gene interaction within the lists of differentially expressed genes provides additional details (Table 2, complete lists of significant networks in Supplemental Tables 2, 3, and 4 inside Additional File 1). The overall pattern of changes with aging and lifestyle in seal skeletal muscle shows similarity to heart development and involves profound changes in cytoskeleton structure and energy metabolism. Table 2 shows the overview of differentially expressed networks of genes in most distant groups of newborn and adult seals. Comparison of similar tables for newborn versus juvenile and juvenile versus adult seals indicates that most dramatic changes in muscle metabolism happen between juvenile and adult ages and associated with adaptation to deep diving rather than aquatic life itself. Using our previous physiological studies to interpret our current data, we would have expected the greatest differences in gene expression to occur between the adults and the other two age classes. Based on the Venn diagram, that is exactly what we found. The largest number of differentially expressed genes was found between the adults and pups, followed by the adults and juveniles and finally the smallest number of differentially expressed genes between the pups and juveniles. Figure 3 shows a different aspect of the age and lifestyle-related differences. When different ages are compared by using the set of pathways statistically over-represented in each of the three lists, the thesis of quantitative differences is corroborated. The set of pathways in all ages is also similar, although there is a difference in significance as estimated by GeneGo Metacore. The *p*-value shown in the logarithmic scale in Figure 4 is an indicator of the signal-to-noise ratio in detecting the activity of the pathway as an entity. Assuming that the noise in all our age-to-age comparisons has the same technical nature (i.e. it comes from the same sample preparation and the same type of microarray) and scale, the over-representation of the *p*-value can be used to estimate the relative activity of the pathway. Subsequently, from the diagram in Figure 4 it follows that certain pathways are significantly over-represented in all age groups, while others differ in activity between pups, juveniles, and adult seals. The complete diagram of significant pathways is given in Supplemental Figure 3 (inside Additional File 1).

**Table 2:** Analysis of interconnected groups (pathways) within the list of genes differentially expressed between adult and newborn Weddell seals.

N	Network	GO Processes	Total nodes	Root nodes	p-Value
1	MLC2, MEF2C, Fibrillin 1, Beta-catenin, VEGF-A, NCOA6 (TRBP)	heart development (18.9%; 2.656e-35), organ development (39.3%; 5.404e-24), anatomical structure morphogenesis (30.4%; 3.790e-22), system development (43.2%; 4.958e-21), developmental process (50.0%; 2.940e-20)	291	6	0

2	NLK, TCF7L2 (TCF4), Beta-catenin, ITF2, VEGF-A, Calmodulin	cellular macromolecule metabolic process (60.5%; 9.000e-22), translational elongation (8.3%; 1.114e-21), macromolecule metabolic process (62.2%; 8.712e-19), cellular metabolic process (69.1%; 1.001e-18), translation (10.8%; 3.135e-15)	392	6	0

3	RanBPM, Tubulin alpha, CLIP170, Tubulin (in microtubules)	mitotic cell cycle (76.7%; 6.661e-30), M phase (73.3%; 2.818e-29), mitosis (63.3%; 2.117e-27), nuclear division (63.3%; 2.117e-27), M phase of mitotic cell cycle (63.3%; 3.441e-27)	30	4	7.09E-05

4	Karyopherin beta 1, Tubulin alpha, CLIP170, Tubulin (in microtubules)	mitotic cell cycle (76.7%; 6.661e-30), M phase (73.3%; 2.818e-29), cell cycle phase (73.3%; 2.038e-26), cell cycle process (76.7%; 9.582e-26), mitosis (60.0%; 2.193e-25)	30	4	7.09E-05

5	COL1A1, Jagged1, HEY1, G-protein alpha-s	translational elongation (10.6%; 4.189e-19), skeletal system development (16.5%; 1.262e-18), embryonic development (22.0%; 4.330e-18), organ morphogenesis (24.3%; 8.874e-18), anatomical structure morphogenesis (30.3%; 2.059e-17)	232	4	0

6	NCOA6 (TRBP), Fibrillin 1, VEGF-A, MLC2	heart development (14.0%; 3.117e-22), translational elongation (9.2%; 8.528e-21), cellular macromolecule metabolic process (59.2%; 3.169e-16), cellular metabolic process (68.5%; 1.129e-14), organ development (32.5%; 1.235e-14)	304	4	0

7	DDX5, Beta-catenin, Collagen III, ZO-1	translational elongation (8.1%; 2.264e-21), cellular macromolecule metabolic process (59.3%; 2.424e-20), cellular metabolic process (69.3%; 1.710e-19), macromolecule metabolic process (60.6%; 5.544e-17), translation (10.8%; 1.321e-15)	396	4	0

8	KIF5B, Tubulin alpha, Tubulin (in microtubules)	microtubule-based movement (58.3%; 1.474e-12), microtubule-based process (66.7%; 5.924e-12), cytoskeleton-dependent intracellular transport (50.0%; 6.967e-12), intermediate filament-based process (41.7%; 2.715e-11), microtubule-based transport (41.7%; 4.662e-11)	12	3	8.55E-05

9	PYGM, IRS-2, G-protein alpha-s	energy reserve metabolic process (23.3%; 1.472e-15), protein amino acid phosphorylation (44.2%; 2.100e-14), phosphorylation (46.5%; 4.698e-14), phosphate metabolic process (48.8%; 1.036e-13), phosphorus metabolic process (48.8%; 1.053e-13)	53	3	4.87E-17

10	Nucleophosmin, Jagged1, VEGF-A	cellular metabolic process (72.2%; 1.602e-08), RNA splicing, via transesterification reactions with bulged adenosine as nucleophile (9.6%; 3.962e-08), nuclear mRNA splicing, via spliceosome (9.6%; 3.962e-08), cellular macromolecule metabolic process (60.9%; 4.590e-08), RNA splicing, via transesterification reactions (9.6%; 5.227e-08)	121	3	1.8E-223

**Figure 5:**
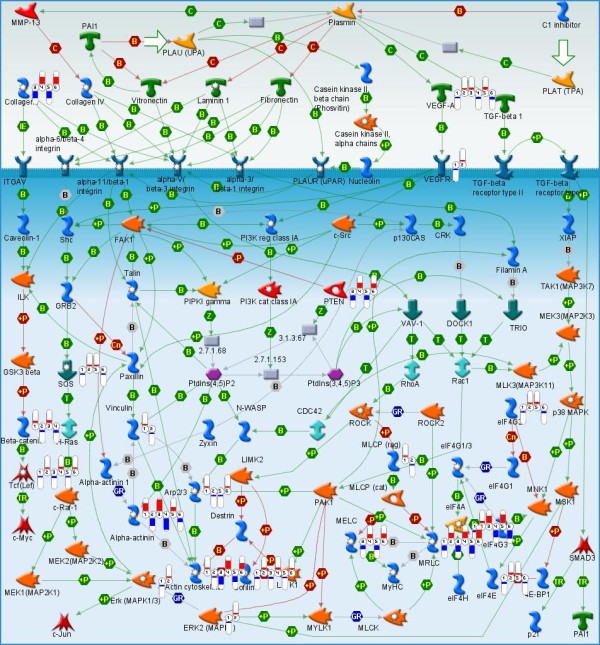
**Cytoskeleton remodeling**. Flags indicate the direction (red for elevated and blue for lowered expression) and relative change. Pairs of flags are attached to genes present and differentially expressed as estimated by J5 selection. Flags 1-2 correspond to comparison between adults and juveniles (AJ); flags 2-3 correspond to comparison between adults and pups (AP); flags 5-6 correspond to comparison between pups and juveniles (PJ). The GeneGo map legend is given in Additional File 1 (Supplemental Figure 5).

Most of the top ten pathways charted in Figure 4 are different variations of the same Cytoskeleton remodeling pathway. A general overview of the cytoskeleton remodeling is given in Figure 4 (see also map legend in Additional File 1). Based on our previous studies, we found a significant shift in muscle fiber type, mitochondrial density, and aerobic efficiency as seals switch from being a non-diving pup to a deep long duration diver as an adult. For these reasons we expected to have significant coverage of the cytoskeleton remodeling pathway, as well as the cardiac hypertrophy pathway, due to the importance of calcium signaling and the calcineurin pathway in driving the majority of these adaptations. Other pathways can be attributed to either the effects of hypoxia (such as changes in glycolysis/glyconeogenesis) or a general re-orientation of the energy metabolism. One of the interesting observations is that changes in seal skeletal muscle show a significant similarity, and have a statistically significant correlation to, the changes documented for human heart hypertrophy. A map of the gene interactions implicated in human heart hypertrophy, overlapped with the pattern of gene expression changes observed in Weddell seals, is given in Supplemental Figure 4 (inside Additional File 1). Like on Figure 5, individual differentially expressed genes are marked with indicators of scale and direction of change. This map is one of the disease pathway template maps from the GeneGo Metacore database.

Our study demonstrates that, in spite of the sacrificed specificity, distant and even cross-species hybridization reflects changes in muscle physiology. The cross-species application of expression microarrays is equivalent to the application of poor quality microarrays. However, the loss of specificity for individual probes and whole probesets can be compensated for by analysis of the biological pathways. Considering genes jointly in the context of the interaction, as opposed to the more traditional independent 'one gene at a time' approach, mitigates the effect of the missing probes and poor hybridization. Our analysis of how gene expression patterns change with the age and lifestyle of Weddell seals has identified major restructuring in cytoskeleton, calcium signaling, and major metabolic pathways. It seems possible that further studies on metabolic and structural transformation of muscle in seals may provide valuable insight into human muscle disorders.

This study is an ongoing project, and we are at the very beginning of the process. Further work will be needed to elucidate the fine species-specific details of gene interaction and to pinpoint the switches guiding the process of the metabolic and structural transformation of skeletal muscle. With more sample studies we might be able to make more quantitative estimations to replace the coarse-grained patterns which provide rough indications of the direction of change and the groups of genes/molecular functions/pathways affected. The comparative analysis of dynamic changes in transcription patterns creates the potential for new research on the evolution of large taxons, thereby branching into a radically different habitat. The most important lesson in methodology from our study is that the combination of genomics, physiology, systems biology, and computational analysis is capable of overcoming the shortcomings of each individual methodology when used in isolation and is able to produce viable results.

## Authors' contributions

SK has performed sample collection, preparation and microarray hybridization as well as biological interpretation of the results. AP has performed microarray data analysis, methodology development, programming and pathway analysis. AS has conducted Western blot experiments. AP and SK have written the paper.

## Supplementary Material

Additional file 1 **Supporting materials**. This is an archive file containing Supplemental Tables 1 and 2 and Supplemental Figures 1 to 5. Click here for file
