# Supplementary figures and images for: Transformation of metabolism with age and lifestyle in Antarctic seals: a case study of systems biology approach to cross-species microarray experiment
Source: BMC Syst Biol. 2010 Sep 29;4:133. doi: 10.1186/1752-0509-4-133 (PMC2958164; doi:10.1186/1752-0509-4-133)

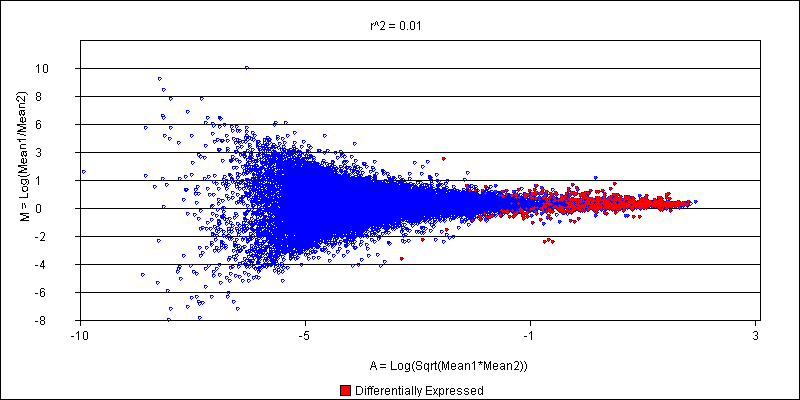

Supplement: Additional file 1 — Supporting materials. This is an archive file containing Supplemental Tables 1 and 2 and Supplemental Figures 1 to 5. [file 1752-0509-4-133-S1.ZIP › Supplfigure2 MA plot J5.gif]

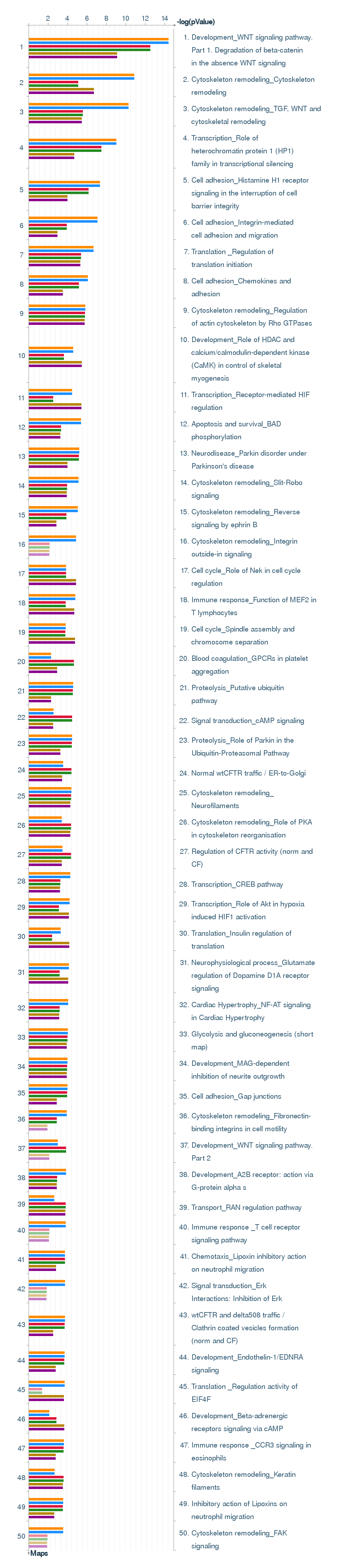

Supplement: Additional file 1 — Supporting materials. This is an archive file containing Supplemental Tables 1 and 2 and Supplemental Figures 1 to 5. [file 1752-0509-4-133-S1.ZIP › SupplFigure3 distribution.png]

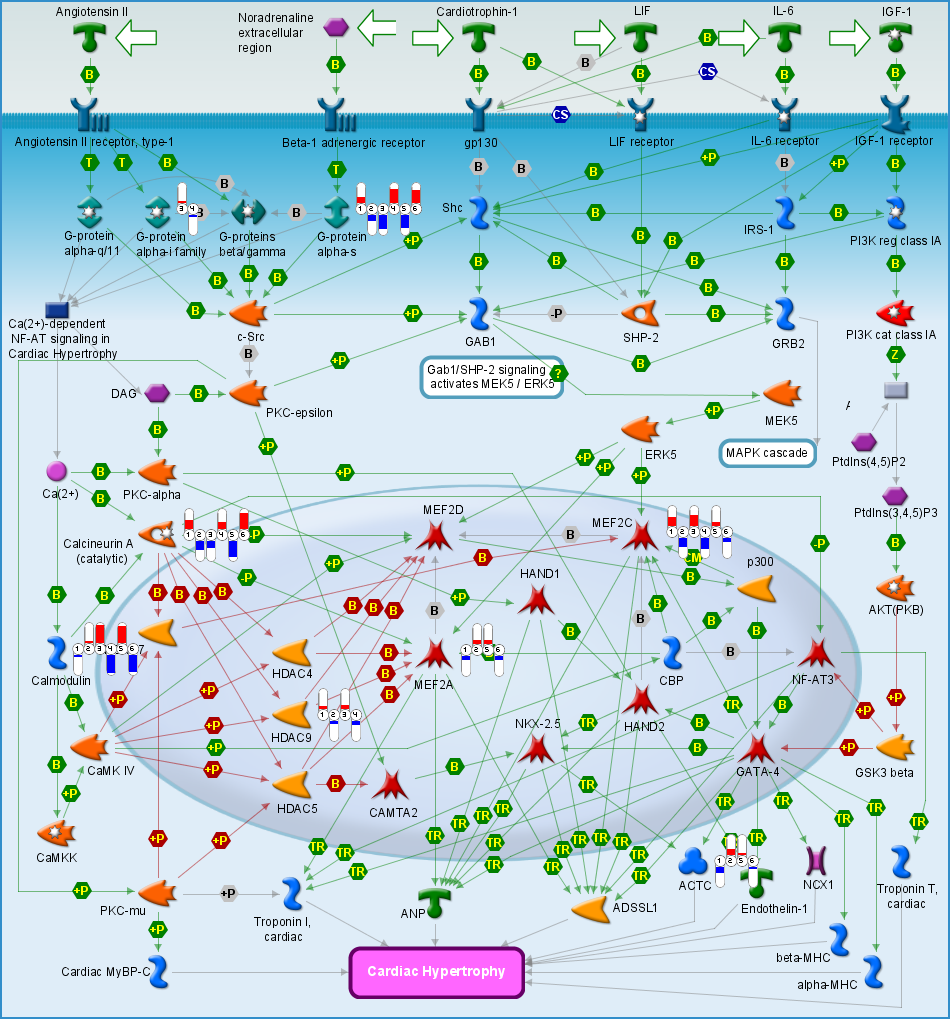

Supplement: Additional file 1 — Supporting materials. This is an archive file containing Supplemental Tables 1 and 2 and Supplemental Figures 1 to 5. [file 1752-0509-4-133-S1.ZIP › SupplFigure4 NF-AT signaling in cardiac hyprtrophy.png]

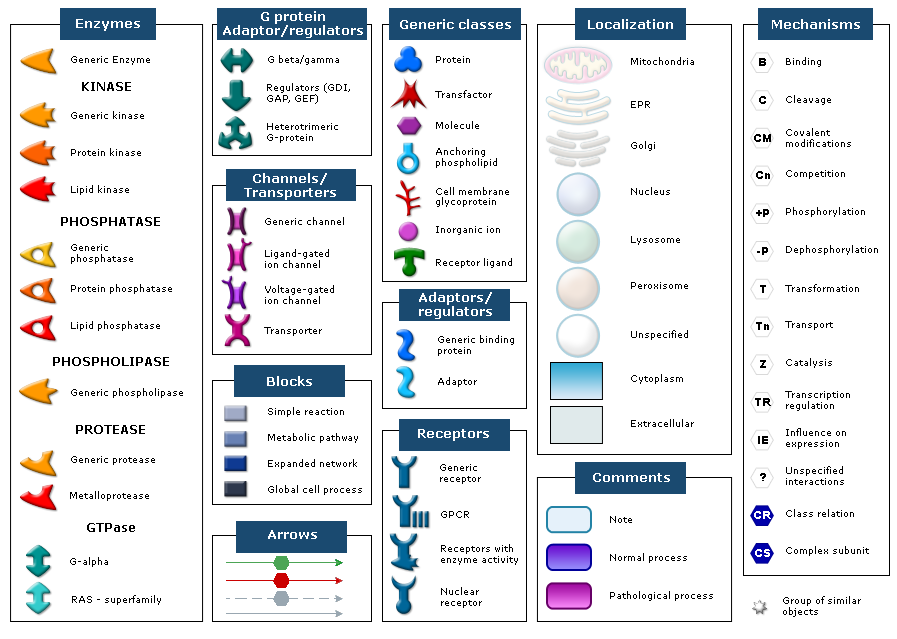

Supplement: Additional file 1 — Supporting materials. This is an archive file containing Supplemental Tables 1 and 2 and Supplemental Figures 1 to 5. [file 1752-0509-4-133-S1.ZIP › SuppFigure5 Metacore Map Legend.PNG]

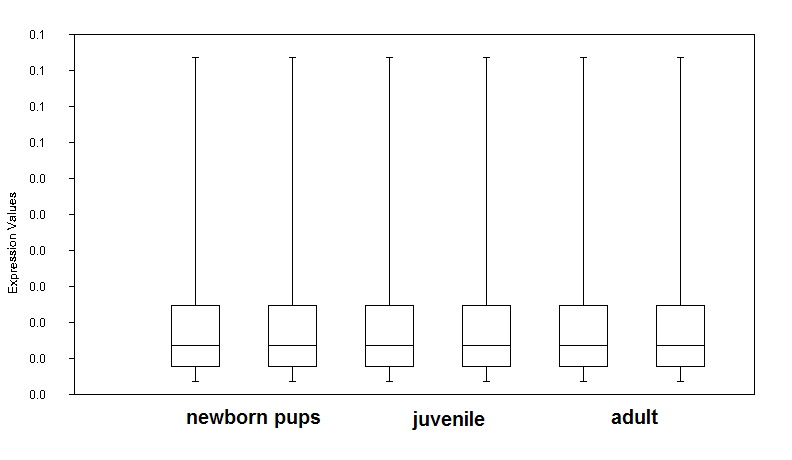

Supplement: Additional file 1 — Supporting materials. This is an archive file containing Supplemental Tables 1 and 2 and Supplemental Figures 1 to 5. [file 1752-0509-4-133-S1.ZIP › SupplFigure1 Box plot of normalized expression.png]
